# Exploring the impact of physical exercise on mental health among female college students: the chain mediating role of coping styles and psychological resilience

**DOI:** 10.3389/fpsyg.2024.1466327

**Published:** 2024-11-18

**Authors:** Longan Cao, Xiaorong Ao, Zhirong Zheng, Zhengban Ran, Jun Lang

**Affiliations:** ^1^School of Physical Education, Southwest University, Chongqing, China; ^2^Teaching and Research Association, Liupanshui Middle School, Guizhou, China

**Keywords:** physical exercise, female college students, coping styles, psychological resilience, mental health

## Abstract

**Background:**

Female college students are a high-risk group for psychological conflicts, and they are more likely to adopt negative coping styles under stress, which can lead to anxiety, depression, and other psychological problems, as well as pose hidden threats to the healthy development of school education and social work. Although physical exercise is an important means of effectively alleviating the psychological health of female college students, and there may be a close relationship between coping styles and psychological resilience with physical exercise and psychological health, specific ways to promote the psychological health of female college students by influencing their coping styles and enhancing their psychological resilience have yet to be explored.

**Methods:**

The present study employed a cross-sectional design to investigate a sample of Chinese undergraduate female college students. A total of 659 participants were recruited during the second half of the 2023–2024 academic year, and they all completed assessments including the Physical Activity Rating Scale, College Mental Health Scale, Brief Coping Styles Questionnaire, and Psychological Resilience Scale. Subsequently, descriptive statistics were utilized to analyze the obtained reliable data.

**Results and conclusion:**

The study revealed two significant correlations among female college students, namely the associations between physical exercise, coping styles, psychological flexibility, and mental health. Specifically, physical exercise exhibited a positive relationship with positive coping styles and psychological resilience while displaying a negative association with negative coping styles and mental health. Moreover, psychological resilience demonstrated a positive correlation with positive coping styles but displayed negative relationships with both negative coping styles and mental health. Additionally, there was a negative relationship observed between positive coping styles and mental health. Furthermore, it was found that physical exercise significantly impacted the mental health of female college students in a detrimental manner; meanwhile, coping styles and psychological resilience played crucial mediating roles in linking physical exercise to mental health outcomes. Ultimately, our mediation model sheds the underlying mechanisms through which physical exercise predicts mental health levels among female college students; these findings underscore the importance of promoting physical exercise as an effective means to enhance overall well-being.

## 1 Introduction

Good mental growth encompasses achieving developmental and emotional milestones, acquiring healthy social skills, learning positive coping mechanisms, and effectively managing setbacks. In contrast, individuals with poor mental health may exhibit deficiencies in academic performance, behavioral regulation, or emotional stability, leading to distress and difficulties in daily life (Torio et al., 2015). Previous research has indicated that female college students exhibit higher levels of mental health issues compared to their male counterparts (Pierceall and Keim, 2007; Brougham et al., 2009), primarily stemming from academic workload and familial pressures (Tang et al., 2018). Academic stress is mainly derived from heightened sensitivity toward experiments and papers that fail to meet expectations (Levecque et al., 2017), while dissatisfaction in personal lives contributes to the accumulation of anxiety. Female college students belong to a transitional group between student life and future career or homemaking responsibilities, thus exacerbating conflicts arising from multiple roles and subsequently intensifying their mental health problems (Yuan et al., 2009). Consequently, chronic anxiety and depression can significantly impact students with physical and mental impairments by diminishing their academic achievements, reducing their quality of life, straining relationships, and causing other issues (dos Santos et al., 2016; WHO, 2021; Mohamed et al., 2024). Moreover, this condition poses a particular challenge for college students as it jeopardizes the conducive development of their environment (Robins et al., 2017). Taken together, these issues underscore the imperative for continuous research endeavors aimed at promoting the prevention and amelioration of mental illnesses, thereby circumventing detrimental consequences at individual, institutional, and societal levels. This is of significant importance in alleviating the internal conditions of female college students under psychological pressure.

Previous studies have identified two strategic objectives for the prevention and mitigation of mental health issues among college students. One objective is to enhance the efficacy of mental health training services provided by educational institutions (Berzin et al., 2011; Solomon et al., 2012; Song et al., 2024), while the other is to cultivate positive personal attributes, such as heightened health consciousness and a sense of life’s purpose (López-Benavente et al., 2018; Zhang L. et al., 2024; Zhang J. R. et al., 2024; Guo et al., 2024). Despite extensive discussions on the correlation between negative emotions, life stress, sleep deprivation, and mental well-being in existing literature (Chang, 2006; Tartar et al., 2015; Li et al., 2022), no scholars have yet explored the relationship between physical activity, coping mechanisms, psychological resilience, and mental health specifically among female college students. Therefore, this study aims to address this research gap.

The term “physical exercise” refers to structured and repeatable physical activities that are designed to improve physical fitness and promote overall health (Caspersen et al., 1985). Such engagement in physical activity not only enhances sleep quality and alleviates the physical and mental fatigue induced by stress and anxiety (Mu et al., 2024), but also boosts workplace performance (Leavell et al., 2019). Since each of these gains can contribute to the development of good mental health, several studies have directly investigated the impact of physical exercise on it. For instance, certain studies have demonstrated that engaging in high-intensity physical activity is effective in reducing the incidence of depression (Munz et al., 2021). Another study revealed that low-intensity aerobic dance exercise can effectively enhance self-esteem and mental health levels among various populations, including women, female physicians, and other demographic groups (Johar et al., 2015; Arfanda et al., 2022; Liu et al., 2022). Furthermore, it has been shown to alleviate symptoms of anxiety in adults (Johar et al., 2012). Although schools are recognized as ideal settings to promote physical exercise, reduce poor mental health and increase adolescent well-being (Morton et al., 2015; ISPAH, 2020), and the significant increase in academic research on interest and participation in women’s sports (Beisecker et al., 2024), a paucity of evidence exists that synthesizes the impact of typical school provision of physical education, physical activity and sports. Importantly, the school students are facing mental health issues, and their performance is not improving in China (Wang et al., 2021). Therefore, this study examined the effects of physical exercise on mental health in a sample of Chinese female college students.

While physical exercise has the potential to directly enhance the mental health of female college students, it is crucial to consider whether other variables mediate this occurrence. Previous studies have tentatively concluded that coping styles play a significant mediating role in the relationship between physical exercise and psychological well-being among college students (Haycock et al., 2020). Intuitively, coping represents an individual’s fundamental response to stress, involving cognitive and behavioral efforts to appropriately address problems when external or internal demands exceed their capacity (Bolger, 1990). The development of relevant theoretical frameworks reflects that students’ mental health predicts their choice of coping styles (Yang et al., 2022). Therefore, considering the positive impact of coping styles on students’ mental health, physical exercise can be utilized as a means to promote positive coping strategies. For instance, evidence suggests that college students who regularly engage in physical exercise demonstrate greater initiative in dealing with stress and challenges (Zhang L. et al., 2024; Zhang J. R. et al., 2024). Consequently, positive and rational coping styles are typically more effective in alleviating negative emotions and reducing the psychological burden associated with stress (Zhou et al., 2019).

In the context of campus life, college students inevitably encounter stress and setbacks that require a strong psychological resilience to foster personal growth. Within this framework, psychological resilience is defined as individuals’ capacity to adapt positively to life circumstances and restore favorable conditions even in the face of adversity and stressful events (Fletcher and Sarkar, 2013; Sisto et al., 2019). Existing evidence suggests that psychological resilience may play a pive in promoting mental health by exhibiting positive associations with levels of psychological well-being and acting as a protective factor in mitigating the direct relationship between perceived stress and psychological distress (Yamada et al., 2017; Lara-Cabrera et al., 2021). Meanwhile, the level of physical exercise among college students seems to be a variable factor that predicts their psychological resilience. Participating in moderate physical activities can effectively improve the psychological resilience and mental well-being of college students (Belcher et al., 2021), thereby positively contributing to the development of mental toughness among female college students during challenging times.

Although the literature suggests that coping modes and resilience independently influence the relationship between physical exercise and mental health, these factors may also be linked together. Indeed, many studies have focused on this connection. For instance, individuals who employ positive coping strategies demonstrate effective problem-solving skills, thereby enhancing their psychological resilience (Laird et al., 2019). Case studies have also illustrated that the utilization of positive coping mechanisms not only fosters favorable growth in psychological resilience but also acts as a moderator for the impact of other cognitive-behavioral factors on psychological resilience (Zhang et al., 2023). Additionally, heightened levels of psychological resilience contribute to emotional regulation, adaptability, and flexibility among female college students, facilitating the adoption of more adaptive coping styles to address challenges and ultimately improving their overall psychological well-being (Wu et al., 2020). As such, it is reasonable to suggest that physical exercise can influence psychological resilience and subsequently impact mental health by modulating coping styles.

Demographic variables (e.g., grade, specialty, place of origin, and whether they are only children or not) have been extensively examined in previous mental health research (Khumalo et al., 2011). However, current studies primarily focus on secondary school students, left-behind children, and college students with limited attention given to female college students. Therefore, this study specifically concentrates on the mental health of female college students. In summary, we constructed a mediation hypothesis model to examine diverse mechanisms through which physical exercise impacts mental health. In this regard, our aim is to incentivize university campus administrators to provide effective exercise strategies for enhancing the mental well-being of female college students. We established physical exercise as the independent variable, mental health as the dependent variable, and coping style and psychological resilience as mediating variables among female college students.

Based on this, we posited the following hypothesis: (1) physical exercise can enhance the mental well-being of female college students; (2) coping styles act as a mediator in the relationship between physical exercise and mental health among female college students; (3) psychological resilience mediates the impact of physical exercise on the mental well-being of female college students; (4) coping styles and psychological resilience as a chain mediating the effects of physical exercise on the mental health of female college students.

## 2 Materials and methods

### 2.1 Participants and procedure

The survey employed the principle of random sampling to select female college students from Chongqing universities as the target population. Freshmen and sophomores completed questionnaires during their respective physical education classes, which were administered by instructors and collected on-site. The standardized online forms will be completed by a randomly selected sample of junior and senior students. A total of 700 female college students agreed to participate in our study. With typical completion rates, it took approximately 20 min for participants to fill out the questionnaires. After excluding missing data and invalid responses, the final sample consisted of 659 participants (94.14%), The participants’ age ranged from 18 to 23 years, with a mean age of 19.30 years; categorized by academic year, there were 198 freshmen, 207 sophomores, 119 juniors, and 135 seniors; based on place of origin, there were 385 rural students and 274 urban students; with regard to majors, there were 412 social sciences majors and 247 natural sciences majors.

Informed consent was obtained from all participants for this survey, and the research team provided them with corresponding souvenirs upon completion. This survey protocol has been approved by the Ethics Committee of Southwest University Human and adheres to the Helsinki Declaration of Ethical Standards.

### 2.2 Materials

#### 2.2.1 Physical exercise

We selected the revised Physical Activity Rating Scale-3 (PARS-3) due to its robust reliability and validity in a Chinese school context (Liang, 1994; Lin et al., 2022). This scale evaluates physical exercise engagement among female college students across three dimensions: intensity, duration, and frequency, with each dimension rated on a 5-point scale (1–5). The score for physical activity participation is calculated as intensity multiplied by (duration minus one) multiplied by frequency, resulting in a score ranging from 0 to 100. A score of ≤19 indicates low exercise level, while scores between 20 and 42 indicate medium exercise level, and scores ≥43 indicate high exercise level. This scale has been proven to be reliable in previous experiments (Duan et al., 2022; Fu et al., 2023), and in the current study, it demonstrates good validity without any modifications. The average values for activity intensity, duration, and frequency are 3.35, 3.67, and 2.82 respectively (SD=0.89, 0.72, 0.86). The Cronbach’s alpha coefficient is 0.78, indicating that participants actively engage in daily exercise.

#### 2.2.2 Coping styles

The Simplified Coping Styles Questionnaire (SCSQ), a 20-item scale developed by Xie (1998), was selected to evaluate an individual’s coping strategies and methods for managing stress and difficulties. A 4-point scale (0 = not used, 3 = often used) was employed. The total score for positive coping ranged from 0 to 36; higher scores indicated more effective coping mechanisms. The scale has previously been validated and demonstrates robust reliability and validity (Chen et al., 2018; Miao et al., 2021). In this study, the questionnaire demonstrated good reliability and validity with a Cronbach’s alpha coefficient of 0.88.

#### 2.2.3 Psychological resilience

We chose the Chinese version of the revised CD-RISC scale (Yu and Zhang, 2007), which is widely used to assess individuals’ psychological resilience. This scale consists of 25 items that cover dimensions such as optimism, strength, and resilience. These items are rated on a 5-point scale ranging from “0 = never” to “4 = always,” with higher scores indicating greater psychological resilience. The scale has been validated for reliability and validity in previous experiments with Chinese populations (Wang et al., 2023). In the current study, we formally assessed the scale’s reliability, which yielded a Cronbach’s alpha coefficient of 0.90.

#### 2.2.4 Mental health

We utilized the Chinese College Student Mental Health Scale (CCSMHS) to evaluate the prevailing mental health issues among Chinese college students and quantify the level of psychological distress experienced by this demographic (Zheng et al., 2005). After consulting with experts from the Department of Psychology and engaging in discussions and exchanges, we obtained test indicators that are aligned with the mental well-being of college students. The consensus among teachers was that the CCSMHS scale effectively measures the level of mental health in this population. Comprising 12 dimensions and a total of 104 entries for psychological symptoms, participants need to rate their frequency of experiencing specific symptoms over the past month on a 5-point scale: 1 = never, 2 = occasionally, 3 = sometimes, 4 = often, and 5 = always; lower scores indicate better mental health status. The scale exhibits excellent reliability and validity, as evidenced by a Cronbach’s alpha coefficient of 0.92, which aligns well with the measurement tools employed for assessing the mental health of Chinese college students.

### 2.3 Data analysis

The data analysis was performed using SPSS 26.0 and the Process 3.5 plugin for SPSS. Firstly, the reliability and validity of the scales measuring exercise level, coping styles, psychological resilience, and mental health among college students were validated through the KMO test and Cronbach’s alpha coefficient. Secondly, a common method bias test was conducted on the sample data to ensure data quality reliability. Thirdly, Pearson’s bivariate correlation analysis and linear regression analysis were employed to examine relationships between all variables. Finally, Model 6 in the process 3.5 plugin program was utilized to explore comprehensively the predictive role of exercise on mental health among female college students while revealing a chain-mediated effect of coping strategies and psychological resilience in influencing mental health through exercise. A self-selected sampling method with 5,000 samples was chosen to obtain robust standard errors along with a 95% bias-corrected confidence interval (CI). If CI does not include zero, it indicates significant mediation effects (DiCiccio and Efron, 1996).

## 3 Results

### 3.1 Common method bias tests

As the data in this study were collected through questionnaire distribution and the survey respondents share a single nature, there is a potential for common method bias. Therefore, we conducted Harman’s single-factor test on the 34 factors extracted from the data and found that the maximum factor variance explained was 30.74%, which is below the critical value of 40% (Podsakoff et al., 2003; Zhou and Long, 2004). Thus, we conclude that no significant common method bias is present in our survey data.

### 3.2 The relationships between physical exercise, coping styles, psychological resilience, and mental health

In this part, the Pearson correlation analysis was employed in this section to thoroughly investigate the associations among the four variables of physical exercise, coping style, psychological resilience, and mental health. As shown in Table 1, all variables exhibited significant pairwise correlations (*p* < 0.01) within the surveyed sample. A noteworthy positive correlation was observed between physical exercise and both positive coping styles and psychological resilience, while a significant negative correlation was found with negative coping styles and psychological well-being. Furthermore, a substantial positive association emerged between positive coping styles and psychological resilience, accompanied by a notable negative relationship with negative coping styles and psychological well-being. Additionally, a marked inverse correlation existed between negative coping styles and psychological resilience, while displaying a significant positive connection with psychological well-being. Lastly, a considerable adverse association was identified between psychological resilience and mental health. Furthermore, there is a significant correlation (*p* < 0.01) between activity intensity, duration, and frequency with physical exercise from various aspects of self-exercise. Among them, the strongest correlation exists between activity intensity and physical exercise. This indirectly confirms the effectiveness of sports activities in alleviating mental health problems.

**Table 1:** Correlation coefficients between the study variables.

Variables	*M*	SD	1	2	3	4	5	6	7	8	9
1 Physical exercise	21.56	11.77	1								
2 Activity intensity	3.35	0.89	0.86^**^	1							
3 Activity time	3.67	0.72	0.78^**^	0.48^**^	1						
4 Motion frequency	2.82	0.86	0.77^**^	0.55^**^	0.43^**^	1					
5 Coping styles	40.68	7.45	0.35^**^	0.25^**^	0.22^**^	0.26^**^	1				
6 Positive coping styles	31.56	6.78	0.52^**^	0.50^**^	0.47^**^	0.56^**^	0.59^**^	1			
7 Negative coping styles	9.12	5.74	−0.41^**^	−0.45^**^	−0.48^**^	−0.48^**^	0.36^**^	−0.55^**^	1		
8 Psychological resilience	79.87	19.17	0.56^**^	0.41^**^	0.46^**^	0.56^**^	0.57^**^	0.88^**^	−0.64^**^	1	
9 Mental health	189.54	35.69	−0.55^**^	−0.67^**^	−0.51^**^	−0.55^**^	−0.42^**^	−0.82^**^	0.87^**^	−0.61^**^	1

In conclusion, the results indicate that female college students with higher levels of physical exercise, positive coping strategies, and psychological resilience tend to have lower scores on the Chinese College Students’ Mental Health Scale (CCSMHS), which reflects good mental health. This indirectly demonstrates the positive impact of physical exercise on individuals’ mental well-being.

In order to further ascertain the relationship between variables, we conducted a collinearity diagnosis experiment on physical exercise, coping strategies, and psychological resilience among female college students. The data reveals that all coefficients for variable conditions are below 30 (4.566, 12.532, 23.583), VIF is less than 5 (1.339, 1.483, 1.822), and tolerance exceeds 0.3 (0.992, 0.894, 0.827), indicating the absence of any collinearity issues and enabling subsequent tests to be carried out.

### 3.3 Mediated effect test: the effects of physical exercise on mental health

**Figure 1:**
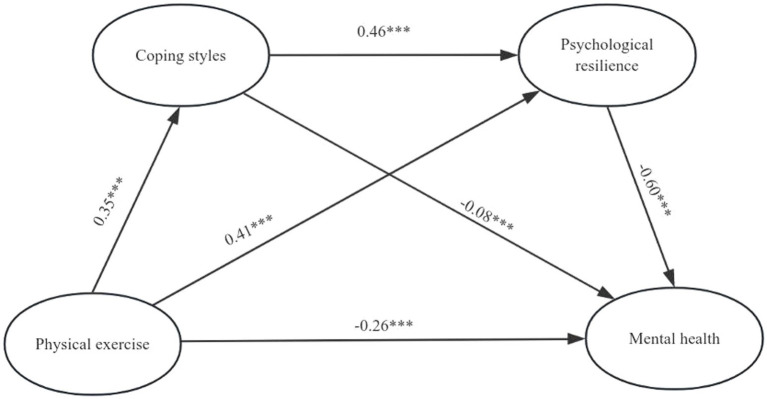
Model showing the mediating roles of coping styles and psychological resilience in the relationship between physical exercise and mental. ***p < 0.001.

**Figure 2:**
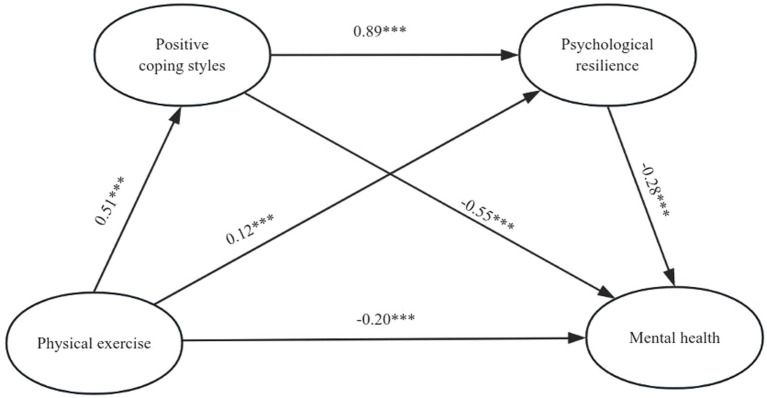
Model showing the mediating roles of positive coping styles and psychological resilience in the relationship between physical exercise and mental. ***p < 0.001.

**Figure 3:**
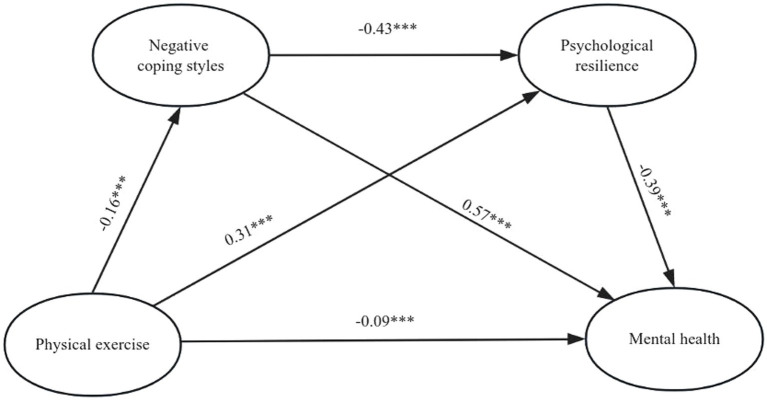
Model showing the mediating roles of negative coping styles and psychological resilience in the relationship between physical exercise and mental. ***p < 0.001.

Based on the previous hypotheses, physical exercise was designated as the independent variable, while mental health served as the dependent variable. Coping styles and psychological resilience were identified as mediator variables. To examine the mediating effects of the overall sample and different coping styles, we employed Model 6 (Process 3.5 plug-in in SPSS) using the Bootstrap method to establish a chain mediation model. The analysis of both the overall sample and samples with different coping styles revealed that physical exercise not only had a significant direct effect on mental health but also demonstrated significant mediation effects through three indirect pathways: path 1 (physical exercise → coping styles [total/positive/negative] → mental health), path 2 (physical exercise → psychological resilience → mental health), and path 3 (physical exercise → coping styles [total/positive/negative] → psychological resilience → mental health). All three paths exhibited noteworthy mediating effects. The details can be found in Table 2. The mediation model paths were derived by combining the results of the sample analyses mentioned in Figures 1–3. The aforementioned findings indicate that the coefficients of each path have reached statistical significance.

**Table 2:** Summary of the mediating effect of physical exercise on the psychological well-being of female college students.

Path	Total			Positive coping style			Negative coping style		
	Effect size	SE	95%CI	Effect size	SE	95%CI	Effect size	SE	95%CI
Total mediating effect	−0.0154	0.0009	−0.0161, −0.0130	−0.0154	0.0009	−0.0161, −0.0130	−0.0154	0.0009	−0.0161, −0.0130
Direct effect	−0.0089	0.0006	−0.0083, −0.0064	−0.0069	0.0006	−0.0063, −0.0044	−0.0079	0.0006	−0.0073, −0.0054
Total indirect effect	−0.0065	0.0008	−0.0088, −0.0065	−0.0085	0.0008	−0.0098, −0.0086	−0.0064	0.0008	−0.0087, −0.0076
Indirect 1	−0.0009	0.0004	−0.0015, −0.0003	−0.0055	0.0007	−0.0065, −0.0062	−0.0017	0.0005	−0.0038, −0.0016
Indirect 2	−0.0043	0.0008	−0.0060, −0.0003	−0.0009	0.0005	−0.0007, −0.0003	−0.0033	0.0007	−0.0028, −0.0009
Indirect 3	−0.0013	0.0004	−0.0013, −0.0009	−0.0021	0.0006	−0.0026, −0.0018	−0.0013	0.0007	−0.0034, −0.0003

Ind1: physical exercise → coping styles [total/positive/negative] → mental health; Ind2: physical exercise → psychological resilience → mental health; Ind3: physical exercise → coping styles [total/positive/negative] → psychological resilience → mental health.

In summary, the findings underscore the significant chain-mediated impact of physical exercise on mental health through its influence on coping styles and psychological resilience. This substantiates that physical exercise not only directly affects mental health but also modulates an individual’s coping strategies and fosters psychological resilience, thereby indirectly influencing overall mental well-being.

## 4 Discussion

This study investigated the mechanistic role of physical exercise in mental health among female college students, using coping style and psychological resilience as mediating factors to construct an intermediate model.

Firstly, we confirmed in this study that physical exercise is a significant predictor of mental health among female college students. Physical exercise can directly and negatively impact the mental health of female college students, this finding aligns with previous research results (Zhang and Min, 2022). Thus, it lends support to the H1 hypothesis.

In conjunction with previous research findings, engagement in physical exercise has been shown to effectively mitigate social withdrawal and aggression among female college students (Liu F. Y. et al., 2024; Liu M. et al., 2024), alleviate negative emotions, enhance frustration coping abilities through exercise-induced pleasure, and foster their social and academic adaptability (De Moor et al., 2006; Conradsson et al., 2010; Fitzgerald et al., 2012; Ligeza et al., 2019). Furthermore, aerobics and basketball exercises are particularly well-suited for the age group of female college students. Deeper analysis can be understood as rhythmic exercises with equivalent intensity demonstrate greater efficacy in alleviating feelings of inferiority compared to non-rhythmic exercises (Zhang and Min, 2022). Hence, it can be inferred that sports have the potential to alleviate tension and promote psychological well-being among female college students. However, when designing physical exercise programs for this population, careful consideration should be given to selecting appropriate sports activities and determining optimal exercise dosage in order to facilitate the accumulation of positive experiences and subsequently promote holistic development by harnessing physical, mental, and social resources.

In addition to examining the direct effects, we also investigated the indirect effects of the relationship between physical exercise and mental health. The study provided evidence for a significant correlation between physical exercise and individuals’ coping styles. Specifically, as the intensity, duration, and frequency of physical exercise increased, individuals were more likely to adopt positive coping styles; conversely, a negative correlation with negative coping styles was observed (Sheng et al., 2018; Liu F. Y. et al., 2024; Liu M. et al., 2024). These findings are consistent with previous research outcomes. Therefore, our findings demonstrate that coping styles play a mediating role in the association between physical exercise and mental health, thus supporting H2. On one hand, engaging in physical exercise aids in boosting metabolism, enhancing oxygen supply to the brain, and stimulating hormones such as dopamine (Berse et al., 2015). This fosters cognitive agility and facilitates effective problem-solving when confronted with challenges. On the other hand, individuals exhibiting positive coping styles demonstrate superior regulation of stress responses (Kocot and Goodman, 2003), thereby assisting in alleviating psychological burdens (Lee et al., 2021). Given limited research on the specific impact of coping styles on mental health issues among female college students, our findings further reinforce the established correlation between these factors. The above points clearly demonstrate that coping styles play a crucial role in bridging the gap between physical exercise and mental health. It is recommended that campuses implement targeted interventions, such as specialized counseling courses, tailored exercise programs, and effective emotional management strategies. These measures can effectively enhance psychological resilience and foster positive coping mechanisms among female university students.

Based on data from indirect pathway 2, the results indicate a significant specific indirect effect of “physical exercise → psychological resilience → psychological well-being.” This suggests that physical exercise not only directly predicts but also indirectly promotes psychological well-being through its mediating role in enhancing psychological resilience (Wiedenman et al., 2023). These findings align with previous research and support hypothesis H3. Previous studies have demonstrated that female college students may be more inclined than male students to rely on physical exercise for boosting their levels of psychological resilience. This difference can be attributed to the fact that male students often associate masculinity and interpersonal styles with sports, which directly contribute to improving their overall mental well-being (Chen et al., 2022). While resilience models for female college students emphasize the importance of promoting factors such as competence, self-efficacy, parental support and adult mentoring to develop resilience, many of these factors are already present in sports environments (Fergus and Zimmerman, 2005). These elements are essential for cultivating exercise-related psychological need satisfaction (PNSE), which includes competence, autonomy and relatedness (Deci and Ryan, 2008). Ensuring a multifaceted and suitable environment is crucial to safeguarding the mental health of female university students. Therefore, resilience, as a protective factor for mental health, can be assessed through sports to gradually increase the level of mental resilience among female college students. This will enable them to better resist corresponding pressures and face challenges and adversities in their lives calmly. Additionally, it can help them subconsciously change negative cognitive styles and effectively regulate their negative emotions, ultimately leading to an improvement in their overall mental health.

Finally, this study revealed the mediating role of coping styles and psychological resilience in the relationship between physical exercise and psychological well-being, thus confirming the validity of hypothesis H4. Although there is no previous direct evidence linking perceptions to physical exercise, coping styles, psychological resilience, and mental health among female college students, several studies have demonstrated that physical exercise has a positive impact on emotions (Brett et al., 2016). When individuals experience positive emotions, they are more likely to make optimistic judgments and choices (Blanchette and Richards, 2010), leading to more effective coping responses. This explains the positive predictive effect of physical exercise on coping styles. Furthermore, individuals with positive coping styles tend to hold favorable attitudes toward life events or academic stressors (Horwitz et al., 2010), thereby moderating mental stress levels and enhancing psychological resilience. Consequently, their psyche becomes more resilient and optimistic (Laird et al., 2019). In conclusion, based on the aforementioned studies, we believe that promoting regular physical exercise among female college students can optimize their coping styles while enhancing psychological resilience and improving stress resistance levels for better overall mental health outcomes. This provides valuable practical insights for addressing mental health issues faced by female college students.

## 5 Limitations and prospects

Based on the correct proof assumed in this article, we suggest encouraging female college students to participate in group sports activities during physical training. This will help foster positive interpersonal relationships and provide a healthy outlet for venting frustrations. In terms of physical education teacher training, efforts should be made to acquire comprehensive knowledge about mental health and purposefully strengthen psychological guidance through various sports activities. As for curriculum arrangements, it is recommended that female college students engage in low-intensity exercises such as recreational volleyball, jogging, or Tai Chi for approximately 1 h per day or participate in moderate- to high-intensity exercises like running, swimming, or tennis 3–5 times a week with sessions lasting around half an hour. In terms of promotional strategies, we should utilize the internet to share diverse examples of extracurricular sports activities and enhance the psychological well-being of individuals across different schools through unique and enjoyable approaches.

Although our study has enhanced the academic understanding of the relationship between physical exercise and mental health among female college students, there are certain limitations. Firstly, in terms of variable selection, this study did not comprehensively encompass numerous factors influencing physical exercise and mental health among female college students. In future studies, a comprehensive analysis can be conducted by expanding the range of variables, introducing new measurement methods or tools, considering both external environmental factors and individual characteristics comprehensively, and integrating existing research findings. For example, based on previous authors’ conclusions, attempts have been made to incorporate mediating variables such as social support, life satisfaction, and self-efficacy to explore the correlations between various factors in order to broaden measures for improving mental health. Secondly, the survey sample in this study only includes a subset of female college students from Chongqing colleges and universities with a small sample size which hampers its ability to fully reflect the overall situation; thus reducing generalizability and analytical efficacy of the findings. In future research endeavors, data selection should be expanded to include more regions and student grades for survey research to ensure diversity and representativeness within the sample. Finally, in terms of research methodology, this study utilized a cross-sectional design to examine the superficial associations among variables. However, potential recall bias might have affected data accuracy and hindered causal inference due to the use of an outdated survey scale that required respondents to recall information during completion. For future studies, we will consider implementing a longitudinal intervention experimental design or incorporating both longitudinal and cross-sectional approaches for an extended follow-up period in order to obtain a more comprehensive understanding of the dynamic relationship between physical exercise and mental health.

## 6 Conclusion

This study investigated the associations between physical exercise, coping styles, psychological resilience, and mental health in female college students. Physical exercise demonstrated a positive correlation with positive coping styles and psychological resilience while showing a negative correlation with negative coping styles and mental health. Psychological resilience exhibited a positive association with positive coping styles and a negative association with negative coping styles as well as mental health. Additionally, positive coping styles displayed a negative relationship with mental health. The findings of this study also confirmed that physical exercise significantly impacted the mental health of female college students in an adverse manner. Furthermore, it was observed that coping style and psychological flexibility played an intermediary role in mediating the effects of physical exercise on mental health.

## Data Availability

The original contributions presented in the study are included in the article/Supplementary material, further inquiries can be directed to the corresponding authors.
