# Supplementary material for: Exploring the impact of physical exercise on mental health among female college students: the chain mediating role of coping styles and psychological resilience
Source: Front Psychol. 2024 Nov 18;15:1466327. doi: 10.3389/fpsyg.2024.1466327 (PMC11610508; doi:10.3389/fpsyg.2024.1466327)
Supplement: Supplementary file 1 [file Data_Sheet_1.docx]

**Questionnaire 1**

Dear Classmate:

Hello! Thank you for your participation in this survey, this questionnaire mainly involves female college students' physical exercise, coping styles, psychological resilience and mental health, the data collected in this survey is only for scientific research, and your answers and personal information will be kept strictly confidential. There is no right or wrong answer, please feel free to answer according to your actual situation and true feelings, your help will help me to complete my thesis, thank you for your support!

In the following questions, you are required to provide some basic personal information. Demographic Scale (please tick as appropriate)

1.Your place of origin: A Urban B Rural

2.Are you an only child: A Yes B No

3.Your specialty category: A Social Sciences B Natural Sciences

4.Your grade level: A Freshman B Sophomore C Junior D Senior

**Questionnaire 2**

**Physical exercise Rating Scale-3 (PARS-3)**

The following questions, measure how physically active you have been in the last month, 5 choices for each question, please choose the appropriate one and fill in the answer number in the ( ) at the end of the question.

1.What is the intensity of your physical activity( )

A. Light exercise (e.g., walking, doing radio gymnastics, playing goalball, etc.)

B. Low-intensity, less-stressful exercise (e.g., recreational volleyball, jogging, tai chi, etc.)

C. More intense sustained exercise at moderate intensity (e.g., cycling, running,table tennis, etc.)

D. High-intensity, but not sustained, exercise that involves shortness of breath and a lot of sweating (e.g., playing badminton, basketball, tennis, soccer, etc.)

E.High-intensity, sustained exercise with rapid breathing and heavy sweating(e.g.,running,aerobics sets,swimming, etc.)

2. How many minutes at a time do you perform physical activity of the above intensity()

A . Less than 10 minutes

B.11 to 20 minutes

C.21 to 30 minutes

D. 31 to 59 minutes

E.60 minutes or more

3. How many times a month do you do the above sports activities ()A. Less than 1 time a month

B. 2 to 3 times a month

C. 1 to 2 times per week

D. 3 to 5 times per week

E. Approximately 1 time per day

**Questionnaire 3**

**Simplified Coping Styles Questionnaire (SCSQ)**

Below is a list of attitudes and practices that you may adopt when you are hit bysetbacks or encounter difficulties in your life. Please read each item carefully and tick thenumber that best suits your own situation.

| **entry** | **not adopt**  **0** | **now and then**  **1** | **resort**  **2** | **Resort**  **3** |
| --- | --- | --- | --- | --- |
| 1.Possible attitudes and approaches when faced with setbacks and blows |  |  |  |  |
| 2. Talks to people and confides his or her inner worries |  |  |  |  |
| 3. Try to see the good side of things |  |  |  |  |
| 4.Change your mind and rediscover what's1mportant in life |  |  |  |  |
| 5. Not taking problems too seriously |  |  |  |  |
| 6.Stand your ground and fight for what you want |  |  |  |  |
| 7.identify several different ways of solving problems |  |  |  |  |
| 8.Seek advice from relatives, friends or classmates |  |  |  |  |
| 9.Changing some of their old practices or some of their own problems |  |  |  |  |
| 10.Draw on the approaches of others who have dealt with similar difficult situations |  |  |  |  |
| 11.Seek hobbies and actively participate in cultural and sports activities |  |  |  |  |
| 12.Try to restrain your disappointment,  remorse,sadness or anger |  |  |  |  |
| 13. Attempted breaks or vacations |  |  |  |  |
| 14. Relieving worries by smoking, drinking, taking pills or food |  |  |  |  |
| 15.Thinks that time will change things and the only thing to do is to wait |  |  |  |  |
| 16.Trying to forget the whole thing |  |  |  |  |
| 17.Relying on others to solve problems |  |  |  |  |
| 18.Accept the reality that there is no alternative |  |  |  |  |
| 19,Fantasize that some miracle might happen to change the status quo |  |  |  |  |
| 20. Comfort yourself |  |  |  |  |

**Questionnaire 4**

**Simplified Coping Styles Questionnaire (SCSQ)**

The following questions 1-25 are the Psychological Resilience Scale. In order to gain a deeper understanding of your psychological resilience before and after exercise intervention, please carefully read each description and select the option that best represents your opinion.

| entry | never  0 | seldom  1 | now and then  2 | non-recurrent  3 | Always  4 |
| --- | --- | --- | --- | --- | --- |
| 1. I am able to adapt to change |  |  |  |  |  |
| 1. I have close and secure relationships |  |  |  |  |  |
| 3. Sometimes fate or God can help. |  |  |  |  |  |
| 1. I can handle whatever happens. |  |  |  |  |  |
| 5. Past successes have given me the confidence  to face challenges |  |  |  |  |  |
| 6. I can see the humor in things |  |  |  |  |  |
| 7. I have become stronger as a result of the trials  and tribulations I have experienced |  |  |  |  |  |
| 8. I consider myself to be a strong person |  |  |  |  |  |
| 9. I believe things always happen for a reason |  |  |  |  |  |
| 1. I will do my best regardless of the outcome |  |  |  |  |  |
| 1. Coping with stress makes me feel empowered |  |  |  |  |  |
| 1. When things seem hopeless, I still persist |  |  |  |  |  |
| 13. I know where to go for help |  |  |  |  |  |
| 1. I am able to concentrate on my thinking |  |  |  |  |  |
| 15. I like to take the lead in solving problems  myself |  |  |  |  |  |
| 1. I will not be discouraged by failure |  |  |  |  |  |
| 17. Coping with stress makes me feel empowered |  |  |  |  |  |
| 18. I can make unusual or difficult decisions |  |  |  |  |  |
| 19. I was able to deal with some unhappiness |  |  |  |  |  |
| 20. I had to go with my gut |  |  |  |  |  |
| 21. I am purposeful |  |  |  |  |  |
| 22. I am in control of my life |  |  |  |  |  |
| 23. I like challenges |  |  |  |  |  |
| 24.I will try my best to reach my goal |  |  |  |  |  |
| 25.I am proud of my achievements |  |  |  |  |  |

**Questionnaire 5**

**Chinese College Student Mental Health Scale (CCSMHS)**

The following quiz items list the problems that we may have. This quiz will not disclose your personal privacy, and there is no right or wrong answer. Please read carefully and select the option that best suits you, then mark it with a “√” in the corresponding bracket.

| **entry** | **hasn't**  **1** | **infrequent**  **2** | **now and then**  **3** | **non-**  **recurrent**  **4** | **Always**  **5** |
| --- | --- | --- | --- | --- | --- |
| 1. Headache or dizziness |  |  |  |  |  |
| 2. Weakness of the body |  |  |  |  |  |
| 3. Doing things must be double-checked |  |  |  |  |  |
| 4. Cannot tolerate their own shortcomings |  |  |  |  |  |
| 5. Worrying about doing things that don't meet expectations |  |  |  |  |  |
| 6. No room for error |  |  |  |  |  |
| 7. Inability to control oneself to do certain things repeatedly,e.g.,constantly checking to see if a door is closed.Constant hand washing, etc. |  |  |  |  |  |
| 8. Unnecessary thoughts or words swirling around in the mind |  |  |  |  |  |
| 9. Feeling of heavy hands or feet |  |  |  |  |  |
| 10. If there is no one to help in times of trouble, you will be at a loss for words |  |  |  |  |  |
| 11. Lack of initiative |  |  |  |  |  |
| 12. Worry that my friends will ignore me |  |  |  |  |  |
| 13. When one has to do something independently, one still wants help |  |  |  |  |  |
| 14. Feeling like you can't handle a lot of things on your  own |  |  |  |  |  |
| 15.Don't know what to do without someone else's plans |  |  |  |  |  |
| 16. If teachers don't assign tasks, they don't know what to  do |  |  |  |  |  |
| 17. Fear of taking the consequences of things |  |  |  |  |  |
| 18. Knowing it's a small thing, but still getting angry |  |  |  |  |  |
| 19. Trying to take advantage |  |  |  |  |  |
| 20. Direct attacks on obnoxious people |  |  |  |  |  |
| 21. Acting on a whim |  |  |  |  |  |
| 22. Can't help hurting people who upset them |  |  |  |  |  |
| 23. Lashing out without regard for the occasion |  |  |  |  |  |
| 24. Doing things without thinking about the  consequences |  |  |  |  |  |
| 25. Inexplicably angry with others |  |  |  |  |  |
| 26. Suspicion of incurable diseases |  |  |  |  |  |
| 27. Feeling that few people understand them |  |  |  |  |  |
| 28. Guilty of sexual longing |  |  |  |  |  |
| 29. Want to curse |  |  |  |  |  |
| 30. Hearing voices that others cannot hear or seeing  things that others cannot see |  |  |  |  |  |
| 31. Feelings of remorse and guilt after masturbation |  |  |  |  |  |
| 32. Doubt that the next person knows what's in my heart |  |  |  |  |  |
| 33. Hate the people around you |  |  |  |  |  |
| 34. Easily fatigued |  |  |  |  |  |
| 35. Feeling bored and fidgety |  |  |  |  |  |
| 36. Oversensitive |  |  |  |  |  |
| 37. Easily stressed |  |  |  |  |  |
| 38. Insomnia |  |  |  |  |  |
| 39. Thinking of things that are too bad to say |  |  |  |  |  |
| 40. Fear of the future |  |  |  |  |  |
| 41. Feeling that everything is difficult |  |  |  |  |  |
| 42. Feeling anxious about something |  |  |  |  |  |
| 43. Getting mad at him |  |  |  |  |  |
| 44. No interest in learning |  |  |  |  |  |
| 45. Trying to be lazy |  |  |  |  |  |
| 46. Difficulty concentrating on work |  |  |  |  |  |
| 47. Chest tightness |  |  |  |  |  |
| 48. Feeling that life is empty and uninteresting |  |  |  |  |  |
| 49. Trying to take advantage |  |  |  |  |  |
| 50. Feeling worthless |  |  |  |  |  |
| 51. On high alert |  |  |  |  |  |
| 52. Feeling unattractive |  |  |  |  |  |
| 53. Suspicion of others hurts me |  |  |  |  |  |
| 54. Feeling like a failure |  |  |  |  |  |
| 55. Nausea |  |  |  |  |  |
| 56. Fear of looking down on oneself |  |  |  |  |  |
| 57. Lack of utilization of one's talents |  |  |  |  |  |
| 58. Feeling like you are always causing problems for  others |  |  |  |  |  |
| 59. No one really cares about me |  |  |  |  |  |
| 60. Feeling that no matter how hard they try they will  never succeed |  |  |  |  |  |
| 61. Fear of attention |  |  |  |  |  |
| 1. want to curse |  |  |  |  |  |
| 63. Feeling shy with the opposite sex |  |  |  |  |  |
| 64. Anxiety about going to parties |  |  |  |  |  |
| 65. Nervous about talking to authority figures |  |  |  |  |  |
| 66. Have the urge to wrestle or destroy things |  |  |  |  |  |
| 67. Nervousness about participating in panel discussions |  |  |  |  |  |
| 68. Blushing when meeting strangers |  |  |  |  |  |
| 69. Difficulty in breathing |  |  |  |  |  |
| 70. Arguing with others |  |  |  |  |  |
| 71. Frustration, depression |  |  |  |  |  |
| 72. Have the urge to hit or hurt others |  |  |  |  |  |
| 73. Feeling uncomfortable in crowds |  |  |  |  |  |
| 74. Easily agitated |  |  |  |  |  |
| 75. It's tempting to hit someone when he or she achieves something. |  |  |  |  |  |
| 76. Jealousy of those who trust more than oneself |  |  |  |  |  |
| 77. Feeling that others love the limelight |  |  |  |  |  |
| 78. Feeling a chill or feverishness |  |  |  |  |  |
| 79. Feeling that sexual behavior is shameful |  |  |  |  |  |
| 80. Thinking of things that are too bad to be true. |  |  |  |  |  |
| 81. Curiosity about the opposite sex makes me feel guilty |  |  |  |  |  |
| 82. Feeling that someone is watching you and talking about you |  |  |  |  |  |
| 83. Think sex dreams are nasty |  |  |  |  |  |
| 84. Feeling that others can control their thoughts |  |  |  |  |  |
| 85. Trying to be lazy |  |  |  |  |  |
| 86. Worried about their hymen breaking or sexual impotence |  |  |  |  |  |
| 87. Feeling of hostility towards others |  |  |  |  |  |
| 88. Doubting their own abilities |  |  |  |  |  |
| 89. Feeling that there is no one you can trust |  |  |  |  |  |
| 90. I am haunted by the insults of others. |  |  |  |  |  |
| 91. Feeling that the goodwill of others is purposeful |  |  |  |  |  |
| 92. Other people did not make appropriate comments on my accomplishments |  |  |  |  |  |
| 93. overly concerned about how others react to me |  |  |  |  |  |
| 94. Unwillingness to listen to others |  |  |  |  |  |
| 95. Get angry when their habits are disrupted |  |  |  |  |  |
| 96. Things must be done very slowly to ensure that they are done correctly |  |  |  |  |  |
| 97. Sexual fantasies prevent me from concentrating on my studies. |  |  |  |  |  |
| 98. Feeling hopeless about the future |  |  |  |  |  |
| 99. Can't help but blame people or things I don't like to see |  |  |  |  |  |
| 100. Feeling depressed |  |  |  |  |  |
| 101. Fear of public speaking |  |  |  |  |  |
| 102. Excessive worry about things that don't really matter |  |  |  |  |  |
| 103. Feeling of being persecuted |  |  |  |  |  |
| 104. Feeling that seminal emission (or menstruation) is dirty |  |  |  |  |  |
